# Impact of Sodium-Glucose Co-transporter 2 Inhibitors After Discharge in Patients With Heart Failure With Reduced Activities of Daily Living: A Single-Center Retrospective Cohort Study

**DOI:** 10.7759/cureus.99009

**Published:** 2025-12-11

**Authors:** Masanori Suzuki, Shintaro Akiyama, Hiroshi Saito, Hiroki Matsui, Ryohkan Funakoshi

**Affiliations:** 1 Faculty of Pharmaceutical Sciences, Teikyo Heisei University, Nakano, JPN; 2 Department of Pharmacy, Kameda Medical Center, Kamogawa, JPN; 3 Department of Rehabilitation, Kameda Medical Center, Kamogawa, JPN; 4 Clinical Research Support Division, Kameda Medical Center, Kamogawa, JPN

**Keywords:** barthel index, frailty, heart failure, readmission, sglt2 inhibitors

## Abstract

Background: Sodium-glucose co-transporter 2 (SGLT2) inhibitors benefit patients with heart failure (HF) with reduced ejection fraction (HFrEF), as well as preserved EFs (HFpEF). Frailty increases the risk of treatment intolerance and adverse events. SGLT2 inhibitors have demonstrated cognitive and clinical benefits, even in cases of severe frailty. However, there is limited evidence regarding its effects post-hospitalization in patients with reduced activities of daily living (ADL). This study investigated whether declining ADL affects SGLT2 inhibitor efficacy in reducing one-year all-cause and HF-specific readmissions.

Methods: This single-center, retrospective cohort study analyzed patients with HF admitted to Kameda General Hospital from December 2020 to February 2022. Eligible patients were discharged without major interventions and were stratified into four groups based on frailty (Barthel Index (BI): < 85) and whether they were prescribed an SGLT2 inhibitor at discharge. Demographic data, comorbidities, laboratory results, medications, and echocardiographic findings were collected. The primary endpoint was one-year HF rehospitalization, while secondary endpoints included all-cause hospitalization and mortality. One-way analysis of variance and chi-square (χ²) tests were used to compare group differences, and logistic regression with inverse probability of treatment weighting (IPTW) was applied to adjust for confounders.

Results: We analyzed 188 patients with HF after excluding 81 based on the eligibility criteria. Patients were stratified into frail (BI < 85, n = 78) and non-frail (BI ≥ 85, n = 110) groups, with the further subdivision based on SGLT2 inhibitor prescription. The frail group was older and had lower renal function, whereas the non-frail group had higher hemoglobin A1c levels and often lived alone. In unadjusted results, SGLT2 inhibitors increased the readmission rate in frail patients. After IPTW adjustment to reduce bias, the results aligned with the unadjusted findings, confirming that frailty and SGLT2 inhibitors significantly influenced outcomes, with a standardized mean difference imbalance in selected variables.

Conclusions: The findings of this study support the cautious use of SGLT2 inhibitors in patients with HF with a BI < 85 at discharge. While these drugs reduced HF rehospitalization in patients with frailty, they also increased the overall rehospitalization rate, likely due to their side effects.

## Introduction

In recent years, as the number of patients with heart failure (HF) continues to increase [1], various treatment options have emerged. Angiotensin receptor-neprilysin inhibitors and sodium-glucose co-transporter 2 (SGLT2) inhibitors have attracted attention as novel therapeutic strategies. Among these, SGLT2 inhibitors, such as dapagliflozin and empagliflozin, have been shown to reduce HF-related events in patients with reduced ejection fraction (HFrEF), regardless of diabetes mellitus status [2,3]. Their efficacy has also been demonstrated in patients with preserved ejection fraction (HFpEF) [4,5], and they are now strongly recommended in treatment guidelines [6]. It has been reported that frailty status is a risk factor for HF exacerbation and death [7]. Patients with frailty because of comorbidities, polypharmacy, or other factors may exhibit lower treatment tolerance and a higher incidence of adverse reactions [8], leading to poor medication adherence and an increased risk of treatment discontinuation. Some clinicians have hesitated to introduce aggressive treatment due to concerns about potential adverse effects [9]. Paradoxically, studies also suggest that patients with diabetic HF and frailty may experience improved cognitive function when treated with SGLT2 inhibitors [10]. Moreover, there is no significant increase in adverse events in HF patients according to the frailty scale [11]. Interestingly, more severe frailty has been linked to greater benefit from SGLT2 inhibitors. However, previous studies have focused on the outpatient population, and evidence is lacking regarding the effect of SGLT2 inhibitors in post-hospitalization HF patients with reduced activities of daily living (ADL). The objective of this study was to stratify hospitalized patients with HF with and without declining ADL to examine whether there is a difference in the degree of the impact of SGLT2 inhibitor administration on readmission (all hospitalizations or HF readmissions) within one year.

## Materials and methods

Methods

Study Design and Setting

This was a single-center, retrospective cohort study conducted by Kameda General Hospital, Kamogawa, Japan. The case examination period was from December 1, 2020, to February 28, 2022. During this period, patients with HFpEF, moderately reduced ejection fraction (HFmrEF), and HFrEF were recruited for observation until February 28, 2023.

Patients

Eligible patients had been admitted with a diagnosis of HF and discharged alive for the first time during the observation period. The exclusion criteria were patients without a Barthel Index (BI) measurement, those who underwent procedures or surgery after admission (coronary revascularization, valvuloplasty/replacement, pacemaker/defibrillator/cardiac resynchronization therapy device implantation, or myocardial ablation), those transferred to other hospitals upon discharge for whom follow-up data were not available, and those who had missing data for weighting.

Exposure

Patients were divided into a frail group with reduced ADL and a non-frail group without reduced ADL. The groups were further divided according to whether an SGLT2 inhibitor was prescribed at the time of discharge. In total, four groups were compared: the frail SGLT2 inhibitor group, the frail non-SGLT2 inhibitor group, the non-frail SGLT2 inhibitor group, and the non-frail non-SGLT2 inhibitor group. The criteria for frailty classification were based on the BI measured by a physical therapist at the time of hospital discharge. The BI consists of 10 questions concerning eating, moving, grooming, toileting, bathing, walking, stair climbing, and dressing, with scores ranging from 0 to 100 (0 = complete dependence, 100 = complete independence). Because BI < 85 has been reported to affect HF rehospitalization [12], patients with BI < 85 were considered frail, and those with BI ≥ 85 were considered non-frail. SGLT2 inhibitor exposure was defined as patients who had been prescribed an SGLT2 inhibitor from the date of admission for HF exacerbation to the date of discharge.

Variables

We collected information on subject demographics, laboratory data, comorbidities, prescription medications, and drug-related data from electronic medical records. Subject attributes were collected as continuous (age, height, and weight) and binary variables (sex, first hospitalization, and living alone). Laboratory data were collected as continuous variables for systolic blood pressure, diastolic blood pressure, pulse rate, estimated glomerular filtration rate (eGFR), hemoglobin A1c (HbA1c), brain natriuretic peptide, serum sodium, serum potassium, hemoglobin (Hb), alanine transferase, total bilirubin, and serum potassium. The percentage of patients with eGFR ≧ 60 mL/min/1.73 m^2^ was classified. Left ventricular ejection fraction (LVEF) was measured at the time of admission for HF exacerbation using the two-dimensional method with transthoracic echocardiography; if the measurement was not performed at the time of admission, the initial measurement was considered the most recent. The following comorbidities were recorded as binary variables: atrial fibrillation/flutter, previous myocardial infarction, type 2 diabetes, hypertension, chronic obstructive pulmonary disease, stroke, cancer, syncope, osteoporosis, and dyslipidemia. The following prescription medications were recorded as binary variables: diuretics (furosemide, azosemide, torasemide, thiazide diuretics, tolvaptan, and spironolactone), angiotensin-converting enzyme inhibitors, angiotensin receptor blockers, angiotensin receptor neprilysin inhibitors, beta-blockers, mineralocorticoid receptor blockers relevant for HF treatment, SGLT2 inhibitors (empagliflozin, dapagliflozin), amiodarone, antiplatelet agents, and anticoagulants. Drug-related information regarding prescription drugs and the number of drugs and doses taken were collected as continuous variables, and the presence or absence of medication guidance was collected as a binary variable.

Endpoints

The primary endpoint was HF rehospitalization within one year, which is defined as hospitalization due to worsening HF during the observation period. The secondary endpoints were hospitalization related to any cause (including planned hospitalizations) and death during the observation period.

Analysis

The differences between groups were subjected to one-way analysis of variance for continuous variables and χ-square tests for categorical variables. Logistic regression weighted by inverse probability of treatment weighting (IPTW) was performed to correct for bias in patient attributes between groups. Factors corrected using IPTW were adjusted for the following confounding factors based on previous studies and clinical findings: age, eGFR, HbA1c, Hb, LVEF, initial hospitalization for HF, and living alone. The magnitude of bias in patient attributes between groups before and after weighting was assessed using the standardized mean difference (SMD). In line with previous studies, SMD was considered highly biased when it exceeded 0.1.

## Results

**Figure 1:**
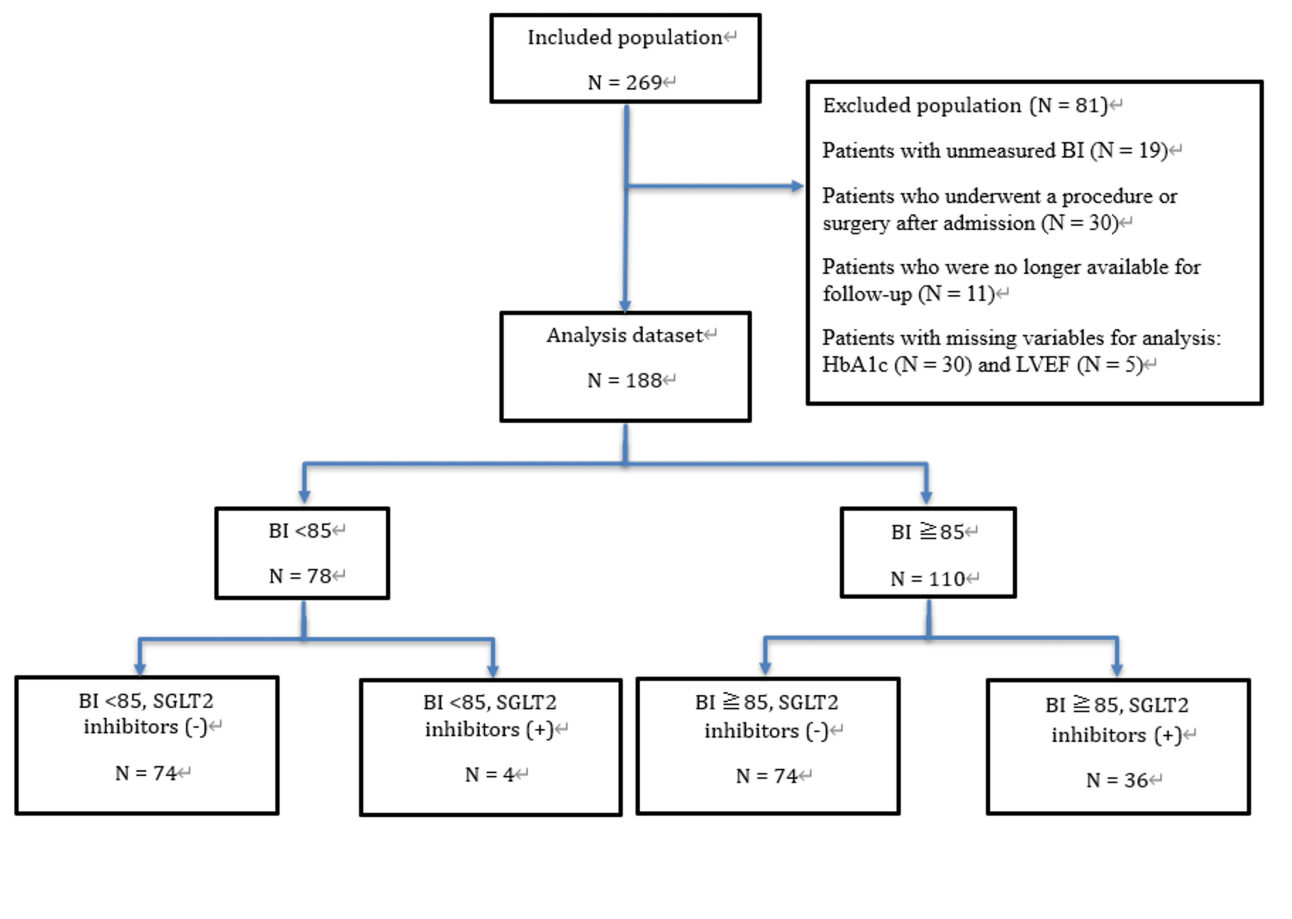
The case-selection flow BI, Barthel Index; SGLT2, sodium-glucose co-transporter 2; HbA1c, hemoglobin A1c; LVEF, left ventricular ejection fraction

The case-selection flow is illustrated in Figure 1. We enrolled 269 patients admitted to Kameda General Hospital with worsening HF during the study period. We excluded 81 patients who met the exclusion criteria (BI not measured, 19 patients; surgery/procedure after admission, 30 patients; unknown outcome, 11 patients; missing variables for analysis, 35 patients), resulting in a total of 188 patients being included in the analysis. Seventy-eight patients had a BI < 85 (74 in the non-SGLT2 inhibitor group and four in the SGLT2 inhibitor group). Of the 110 patients with a BI ≥ 85, 74 were in the non-SGLT2 inhibitor group, and 36 were in the SGLT2 inhibitor group (Figure 1).

Table 1 shows the patient backgrounds between the groups. The non-frail (n = 110) and frail (n = 78) groups tended to be older, with the frail group averaging over 80 years old (p < 0.001). HbA1c levels tended to be higher in the non-frail group of patients prescribed SGLT2 inhibitors than in the other three groups (p = 0.003). The baseline renal function was slightly lower in the frail group than in the non-frail group (p = 0.003). Hb values tended to be higher in the non-frail group than in the frail group (p < 0.001), and EF values tended to be lower in the group prescribed SGLT2 inhibitors than in the group not prescribed SGLT2 inhibitors (p = 0.002). The groups differed in the rate of first HF hospitalization; however, there were numerous cases in which it was not possible to confirm whether it was the first hospitalization (p < 0.001). More than 30% (SGLT2 inhibitors -: 27 (36.5%), SGLT2 inhibitors +: 13 (36.1%)) of the non-frail group lived alone (p = 0.025).

**Table 1:** Baseline characteristics of the study population according to BI *p-value is statistically significant when <0.05. SGLT2i, sodium-glucose co-transporter 2 inhibitor; HF, heart failure; SD, standard deviation; BI, Barthel index; eGFR, estimated glomerular filtration rate; HbA1c, hemoglobin A1c; BNP, brain natriuretic peptide; Hb, hemoglobulin; ALT, alanine transferase; TB, total bilirubin; LVEF, left ventricular ejection fraction; ACEI, angiotensin-converting enzyme inhibitors; ARB, angiotensin receptor blockers; MI, myocardial infarction; COPD, chronic obstructive pulmonary disease; BMI, body mass index; SMD, standardized mean difference

Characteristic	BI <85		BI ≥85		p	SMD	F value^§^ or X-squared^¶^
	SGLT2i		SGLT2i				
	-	+	-	+			
n	74	4	74	36			
Mean age (SD), y	86.72 (7.12)	81.39 (12.72)	75.22 (13.14)	70.67 (13.23)	<0.001*	0.794	21.379^§^
Male, n (%)	40 (54.1)	0 (0)	26 (35.1)	11 (30.6)	0.014*	0.748	10.672^¶^
Physiological measures							
Mean systolic blood pressure (SD), mm Hg	119.95 (18.90)	109.50 (13.00)	115.18 (17.93)	112.83 (20.59)	0.192	0.323	1.5957^§^
Mean diastolic blood pressure (SD), mm Hg	63.97 (10.22)	56.75 (10.75)	66.12 (12.79)	66.19 (13.88)	0.336	0.436	1.1353^§^
Mean pulse rate (SD), beats/min	71.23 (13.55)	71.00 (16.45)	71.00 (11.73)	70.60 (12.78)	0.996	0.023	0.019349^§^
Mean BMI (SD), kg/m^2^	20.06 (4.26)	19.83 (2.50)	21.91 (4.23)	22.72 (4.45)	0.011*	0.45	3.8165^§^
Mean HbA1c (SD) level, %	6.09 (0.98)	6.03 (0.64)	6.11 (1.40)	7.05 (1.84)	0.003*	0.357	4.8154^§^
Mean eGFR (SD), mL/min/1.73 m^2^	38.99 (19.66)	39.92 (8.68)	51.05 (23.72)	50.42 (18.66)	0.003*	0.431	4.7866^§^
eGFR, n (%) ≧ 60 mL/min/1.73 m^2^	10 (13.5)	0 (0.0)	25 (33.8)	7 (19.4)	0.017*	0.541	10.233^¶^
Mean BNP level (SD), pg/mL	432.42 (396.57)	412.10 (257.09)	430.73 (462.29)	442.34 (431.51)	0.999	0.042	0.0095912^§^
Mean sodium level (SD), mEq/L	140.08 (3.67)	136.75 (4.43)	138.62 (3.37)	138.86 (3.23)	0.031*	0.447	3.0268^§^
Mean potassium level (SD), mEq/L	4.32 (0.61)	4.70 (0.22)	4.34 (0.47)	4.37 (0.51)	0.559	0.476	0.69116^§^
Mean Hb level (SD), g/L	11.45 (1.83)	10.88 (0.98)	12.19 (2.74)	13.62 (2.36)	<0.001*	0.743	7.4786^§^
Mean ALT level (SD), U/L	18.32 (14.69)	16.25 (5.38)	29.03 (24.86)	35.39 (48.93)	0.014*	0.435	3.6352^§^
Mean TB level (SD), mg/dL	0.67 (0.38)	0.72 (0.59)	0.83 (0.50)	0.88 (0.66)	0.128	0.229	1.9178^§^
Mean LVEF (SD), %	49.57 (17.28)	37.25 (10.78)	43.81 (19.82)	35.00 (20.08)	0.002*	0.489	5.0843^§^
Medical history, n (%)							
Hospitalization for first-time HF	17 (23.0)	2 (50.0)	36 (48.6)	16 (44.4)	<0.001*	0.456	23.675^¶^
Hospitalization for first-time HF (NA)	46 (62.2)	1 (25.0)	21 (28.4)	9 (25.0)			
Previous MI	5 (6.8)	1 (25.0)	3 (4.1)	2 (5.6)	0.360	0.323	3.2122^¶^
Atrial fibrillation/flutter	27 (36.5)	2 (50.0)	33 (44.6)	14 (38.9)	0.753	0.157	1.1984^¶^
Type 2 diabetes	11 (14.9)	1 (25.0)	10 (13.5)	14 (38.9)	0.009*	0.343	11.544^¶^
Hypertension	37 (50.0)	1 (25.0)	33 (44.6)	21 (58.3)	0.427	0.371	2.781^¶^
COPD	5 (6.8)	0 (0.0)	6 (8.1)	5 (13.9)	0.569	0.307	2.0172^¶^
Stroke	6 (8.1)	2 (50.0)	0 (0.0)	3 (8.3)	<0.001*	0.723	19.839^¶^
Cancer	1 (1.4)	0 (0.0)	9 (12.2)	1 (2.8)	0.030*	0.306	8.9362^¶^
Syncope	0 (0.0)	0 (0.0)	1 (1.4)	0 (0.0)	0.671	0.083	1.5488^¶^
Osteoporosis	2 (2.7)	0 (0.0)	0 (0.0)	0 (0.0)	0.374	0.118	3.1142^¶^
Dyslipidemia	10 (13.5)	0 (0.0)	5 (6.8)	7 (19.4)	0.203	0.401	4.6051^¶^
Treatment, n (%)							
ACEI or ARB	35 (47.3)	0 (0.0)	39 (52.7)	15 (41.7)	0.178	0.745	4.9144^¶^
- ARNI	3 (4.1)	3 (75.0)	5 (6.8)	10 (27.8)	<0.001*	1.082	31.66^¶^
- ACEI/ARB/ARNI	38 (51.4)	3 (75.0)	44 (59.5)	25 (69.4)	0.283	0.286	3.8107^¶^
- β-Blocker	48 (64.9)	2 (50.0)	63 (85.1)	33 (91.7)	0.002 *	0.586	15.201^¶^
Mineralocorticoid-receptor antagonist	21 (28.4)	3 (75.0)	30 (40.5)	28 (77.8)	<0.001*	0.680	25.957^¶^
SGLT2i					<0.001*	NaN	216.26^¶^
- Dapagliflozin	0 (0.0)	4 (100)	0 (0.0)	13 (36.1)			
- Empagliflozin	0 (0.0)	0 (0.0)	0 (0.0)	23 (63.9)			
- Diuretic	67 (90.5)	4 (100)	66 (89.2)	31 (86.1)	0.804	0.299	0.98681^¶^
- Furosemide	58 (78.4)	1 (25.0)	58 (78.4)	23 (63.9)	0.037*	0.671	8.4575^¶^
- Azosemide	5 (6.8)	0 (0.0)	8 (10.8)	5 (13.9)	0.571	0.319	2.0068^¶^
- Torasemide	4 (5.4)	0 (0.0)	0 (0.0)	0 (0.0)	0.098	0.169	6.2961^¶^
- Thiazide	3 (4.1)	0 (0.0)	2 (2.7)	3 (8.3)	0.553	0.242	2.0924^¶^
- Tolvaptan	8 (10.8)	3 (75.0)	14 (18.9)	5 (13.9)	0.006*	0.847	12.458^¶^
- Spironolactone	20 (27.0)	3 (75.0)	25 (33.8)	21 (58.3)	0.005*	0.614	13.029^¶^
- Amiodarone	0 (0.0)	0 (0.0)	3 (4.1)	0 (0.0)	0.195	0.145	4.6966^¶^
- Anti-platelet	2 (2.7)	0 (0.0)	3 (4.1)	0 (0.0)	0.648	0.188	1.6493^¶^
- Anti-coagulation	4 (5.4)	0 (0.0)	10 (13.5)	0 (0.0)	0.055	0.346	7.6174^¶^
Polypharmacy, n (%)	8.08 (3.16)	10.75 (0.96)	7.93 (3.68)	9.39 (3.66)	0.085	0.587	2.2451^§^
Living alone, n (%)	13 (17.6)	0 (0.0)	27 (36.5)	13 (36.1)	0.025*	0.610	9.3267^§^
Mean medication guidance, times (SD)	0.85 (0.66)	1.00 (0.82)	1.12 (0.74)	1.56 (0.97)	<0.001*	0.453	7.0008^§^
Mean Number of doses, count (SD)	2.78 (1.17)	3.00 (0.82)	2.88 (1.35)	2.89 (1.47)	0.958	0.096	0.10322^§^

Table 2 shows the outcomes across groups before adjustment. One-year HF readmission rates in both the frail and non-frail groups tended to be lower when SGLT2 inhibitors were prescribed (p = 0.709). In the frail group, the likelihood of total rehospitalization (37.8%: 28/74 - 100%: 4/4) increased with the prescription of SGLT2 inhibitors, whereas in the non-frail group, the rate of total rehospitalization was lower in the SGLT2 inhibitor group than in the non-SGLT2 inhibitor group (66.2%: 49/74 - 52.8%: 19/36) (p = 0.001).

**Table 2:** Effects of dapagliflozin and empagliflozin on clinical events according to BI *p-value is statistically significant when <0.05. BI, Barthel index; SGLT2i, sodium-glucose co-transporter 2 inhibitor; SMD, standardized mean difference

Variables	BI <85				BI ≥85				P
	SGLT2i				SGLT2i				
	-		+		-		+		
	N = 74		N = 4		N = 74		N = 36		
	N	%	N	%	N	%	N	%	
HF hospitalization ≤1 y	14	18.9	0	0	16	21.6	6	16.7	0.709
All-cause re-admission ≤1 y	28	37.8	4	100	49	66.2	19	52.8	0.001*
All-cause death ≤1 y	15	20.3	1	25	10	13.5	4	11.1	0.526
All-cause death or HF hospitalization ≤1 y	27	36.5	1	25	22	29.7	8	22.2	0.481
All-cause death or all-cause readmission ≤1 y	37	50	4	100	51	68.9	21	58.3	0.041*

To correct for patient background bias among groups, we attempted to correct for confounding factors using multigroup IPTW. Table 3 shows the patient backgrounds of each group after weighting. Patient background bias between the groups was reduced compared to that before weighting. However, the SMD exceeded 0.1 for all groups except for the history of syncope and torsemide, which did not balance among the groups. Table 4 shows the impact of frailty and SGLT2 inhibitors on outcomes, weighted using the above IPTW and adjusted for confounding factors. Both HF readmissions within one year and all readmissions supported the unadjusted results.

**Table 3:** Baseline characteristics of the study population according to BI after IPTW *p-value is statistically significant when <0.05. SGLT2, sodium-glucose co-transporter 2; HF, heart failure; SD, standard deviation; BI, Barthel index; eGFR, estimated glomerular filtration rate; HbA1c, hemoglobin A1c; BNP, brain natriuretic peptide; Hb, hemoglobulin; ALT, alanine transferase; TB, total bilirubin; LVEF, left ventricular ejection fraction; ACEI, angiotensin-converting enzyme inhibitors; ARB, angiotensin receptor blockers; MI, myocardial infarction; COPD, chronic obstructive pulmonary disease; BMI, body mass index; SMD, standardized mean difference

Characteristis	BI <85		BI ≥85		p	SMD
	SGLT2i		SGLT2i			
	-	+	-	+		
n	63.09	39.6	74	62.01		
Mean age (SD), y	78.25 (10.23)	76.72 (10.67)	75.22 (13.14)	75.07 (11.76)	0.761	0.163
Male, n (%)	27.6 (43.8)	0.0 (0.0)	26.0 (35.1)	25.2 (40.6)	0.088	0.636
Physiologic measures						
Mean systolic blood pressure (SD), mm Hg	124.47 (20.68)	112.75 (9.93)	115.18 (17.93)	115.64 (20.55)	0.261	0.333
Mean diastolic blood pressure (SD), mm Hg	66.25 (9.78)	60.22 (11.08)	66.12 (12.79)	65.10 (13.65)	0.744	0.275
Mean pulse rate (SD), beats/min	69.87 (13.58)	73.99 (14.41)	71.00 (11.73)	68.27 (11.69)	0.650	0.234
Mean BMI (SD), kg/m^2^	20.75 (4.29)	20.15 (2.25)	21.91 (4.23)	22.17 (4.00)	0.244	0.332
Mean HbA1c level (SD), %	6.14 (1.12)	5.98 (0.53)	6.11 (1.40)	6.56 (1.01)	0.099	0.302
Mean eGFR (SD), mL/min/1.73 m^2^	47.58 (21.54)	40.46 (6.19)	51.05 (23.72)	47.51 (19.40)	0.013*	0.312
eGFR, n (%) ≧ 60 mL/min/1.73 m^2^	16.9 (26.8)	0.0 (0.0)	25.0 (33.8)	12.0 (19.4)	0.184	0.537
Mean BNP level, pg/mL	271.16 (273.39)	460.23 (232.48)	430.73 (462.29)	405.80 (380.67)	0.049*	0.314
Mean sodium level (SD), mEq/L	139.60 (3.85)	135.96 (4.79)	138.62 (3.37)	138.81 (3.61)	0.437	0.449
Mean potassium level (SD), mEq/L	4.42 (0.62)	4.72 (0.16)	4.34 (0.47)	4.39 (0.61)	<0.001*	0.452
Mean Hb level (SD), g/L	12.23 (2.01)	10.64 (1.16)	12.19 (2.74)	12.77 (2.25)	0.042*	0.566
Mean ALT level (SD), U/L	21.83 (19.67)	17.87 (5.75)	29.03 (24.86)	27.41 (29.13)	0.034*	0.325
Mean TB level (SD), mg/dL	0.66 (0.30)	0.93 (0.70)	0.83 (0.50)	0.71 (0.45)	0.181	0.308
Mean LVEF (SD), %	46.63 (17.87)	40.90 (12.78)	43.81 (19.82)	41.08 (22.27)	0.775	0.184
Medical history, n (%)						
Hospitalization for first-time HF	29.4 (46.7)	16.9 (42.6)	36.0 (48.6)	30.7 (49.5)	0.763	0.328
Hospitalization for first-time HF (NA)	20.7 (32.9)	4.9 (12.5)	21.0 (28.4)	16.6 (26.7)	-	-
Previous MI	1.1 (1.7)	4.9 (12.5)	3.0 (4.1)	1.6 (2.5)	0.246	0.233
Atrial fibrillation/flutter	21.5 (34.0)	22.7 (57.4)	33.0 (44.6)	24.0 (38.7)	0.641	0.259
Type 2 diabetes	18.8 (29.8)	11.8 (29.9)	10.0 (13.5)	24.2 (39.0)	0.471	0.3
Hypertension	31.7 (50.2)	11.8 (29.9)	33.0 (44.6)	38.8 (62.6)	0.475	0.359
COPD	6.4 (10.1)	0.0 (0.0)	6.0 (8.1)	8.1 (13.0)	0.521	0.293
Stroke	4.0 (6.3)	16.8 (42.4)	0.0 (0.0)	4.6 (7.5)	0.037*	0.64
Cancer	0.8 (1.3)	0.0 (0.0)	9.0 (12.2)	1.1 (1.9)	0.039*	0.298
Syncope	0.0 (0.0)	0.0 (0.0)	1.0 (1.4)	0.0 (0.0)	0.822	0.083
Osteoporosis	0.8 (1.2)	0.0 (0.0)	0.0 (0.0)	0.0 (0.0)	0.78	0.079
Dyslipidemia	14.6 (23.2)	0.0 (0.0)	5.0 (6.8)	12.7 (20.6)	0.19	0.47
Treatment, n (%)						
ACEI or ARB	35.3 (55.9)	0.0 (0.0)	39.0 (52.7)	21.7 (35.0)	0.019*	0.83
ARNI	5.5 (8.6)	21.8 (55.1)	5.0 (6.8)	13.1 (21.1)	0.048*	0.663
ACEI/ARB/ARNI	40.7 (64.6)	21.8 (55.1)	44.0 (59.5)	34.8 (56.1)	0.904	0.108
β-Blocker	40.7 (64.4)	22.8 (57.6)	63.0 (85.1)	51.1 (82.4)	0.359	0.387
Mineralocorticoid-receptor antagonist	28.4 (45.0)	27.8 (70.1)	30.0(40.5)	45.6(73.5)	0.256	0.437
SGLT2 inhibitors					NA	NaN
- Dapagliflozin	0.0 (0.0)	39.6 (100.0)	0.0 (0.0)	22.9 (36.9)		
- Empagliflozin	0.0 (0.0)	0.0 (0.0)	0.0 (0.0)	39.1 (63.1)		
- Diuretic	57.8 (91.6)	39.6 (100.0)	66.0 (89.2)	53.1 (85.6)	0.438	0.313
- Furosemide	48.1 (76.3)	4.9 (12.5)	58.0 (78.4)	40.5 (65.2)	0.003*	0.886
- Azosemide	1.9 (3.0)	0.0 (0.0)	8.0 (10.8)	10.2 (16.4)	0.206	0.385
- Torasemide	1.0 (1.5)	0.0 (0.0)	0.0 (0.0)	0.0 (0.0)	0.739	0.088
- Thiazides	1.2 (2.0)	0.0 (0.0)	2.0 (2.7)	3.9 (6.4)	0.448	0.208
- Tolvaptan	3.3 (5.2)	34.6 (87.3)	14.0 (18.9)	6.9 (11.1)	<0.001*	1.332
- Spironolactone	28.0 (44.3)	27.8 (70.1)	25.0 (33.8)	37.2 (60.0)	0.316	0.436
- Amiodarone	0.0 (0.0)	0.0 (0.0)	3.0 (4.1)	0.0 (0.0)	0.592	0.145
- Anti-platelet	1.2 (2.0)	0.0 (0.0)	3.0 (4.1)	0.0 (0.0)	0.623	0.184
- Anti-coagulation	2.7 (4.2)	0.0 (0.0)	10.0 (13.5)	0.0 (0.0)	0.238	0.34
Polypharmacy, n (%)	8.06 (2.68)	10.38 (0.80)	7.93 (3.68)	9.19 (3.44)	<0.001*	0.555
Living alone, n (%)	17.5 (27.8)	0.0 (0.0)	27.0 (36.5)	18.1 (29.2)	0.146	0.538
Mean medication guidance, times (SD)	0.96 (0.58)	1.00 (0.58)	1.12 (0.74)	1.50 (1.01)	0.09	0.359
Mean Number of doses, count (SD)	2.65 (1.19)	3.00 (0.58)	2.88 (1.35)	2.88 (1.31)	0.691	0.165

**Table 4:** Effects of dapagliflozin and empagliflozin on clinical events according to BI after IPTW BI, Barthel index; SGLT2, sodium-glucose co-transporter 2; CI, confidence interval

Variables	Odds ratio	95% CI
HF hospitalization ≤1 y		
BI ≥85, SGLT2 inhibitors (-)	Ref	Ref
BI ≥85, SGLT2 inhibitors (+)	0.432	(0.142–1.321)
BI <85, SGLT2 inhibitors (-)	0.857	(0.213–3.449)
BI <85, SGLT2 inhibitors (+)	0	(0–0)
All-cause re-admission ≤1 y		
BI ≥85, SGLT2 inhibitors (-)	Ref	Ref
BI ≥85, SGLT2 inhibitors (+)	0.617	(0.241–1.581)
BI <85, SGLT2 inhibitors (-)	0.409	(0.147–1.139)
BI <85, SGLT2 inhibitors (+)	34,609,621.567	(10,156,920.556–117,931,995.075)

## Discussion

In this study, we compared the post-discharge outcomes of 188 patients discharged from Kameda General Hospital with worsening HF with and without SGLT2 inhibitor treatment, grouped based on BI status at discharge. HF rehospitalization within one year tended to decrease when SGLT2 inhibitors were prescribed in both the maintenance and reduced ADL groups (non-frail and frail, respectively). In contrast, overall rehospitalization was lower with SGLT2 inhibitors in the non-frail group but higher with SGLT2 inhibitors in the frail group than in the control group. Moreover, when correcting for potential confounding factors using IPTW, the trend in odds ratios did not deviate from the results in the frail group prescribed SGLT2 inhibitors.

A previous study [11] examining HF rehospitalization and cardiovascular death found that SGLT2 inhibitors were effective in patients with HF with and without frailty, did not increase the frequency of adverse events compared to placebo, and were safe to use. The present study showed similar results for HF rehospitalization. Studies of SGLT2 inhibitors in HF have also reported improved outcomes in patients with HFrEF and HFpEF [2-5]. The results of this study are consistent with potentially effective results obtained with the administration of SGLT2 inhibitors, showing that HF-related rehospitalizations are more likely to be avoided if SGLT2 inhibitors are prescribed, regardless of the BI.

A prior study [11] has reported that the occurrence of events due to SGLT2 side effects other than HF, such as dehydration, hypoglycemia, and ketoacidosis, is increased in frail conditions but did not differ between patients with and without treatment with SGLT2 inhibitors. Additionally, the effects of empagliflozin on HF clinical outcomes, as well as renal and quality of life benefits, were consistent regardless of body mass index (BMI), indicating that empagliflozin should not be withheld based on mean BMI or weight loss when administered to patients with HFrEF [13]. However, the increase in overall rehospitalization with the prescription of SGLT2 inhibitors in this study suggests that the increase in hospitalization due to SGLT2 side effects outweighs the rehospitalization-preventive effect of SGLT2 in patients in a frail state with HF.

The results showed a reduction in HF rehospitalizations but an increase in all rehospitalizations. This may be due to the differences in the target populations between the previous and present studies. The population of the previous DAPA-HF post-hoc analysis study included a small proportion of Asians (31.7%), including Japanese individuals; an even smaller proportion (11%) were considered frail [11]. In addition, patients with HF in a frail state included in the previous study were obese (BMI ≥25). Based on this, we believe that the risk of dehydration and other adverse effects due to SGLT2 inhibitors is more pronounced in those in a frail state with a BMI <20, as is the case in Japanese patients. The medical records of all four readmitted cases were reviewed, and three were admitted because of suspected pneumonia; therefore, it was not possible to determine whether there was a direct effect of SGLT2 inhibitors. In contrast, the EMPA-Elderly study [14], which examined empagliflozin in older patients with diabetes, reported that it was well tolerated in terms of safety; this is inconsistent with our results, although the diseases are different. This may have been because the demographics of the case population differed from those of the target population of the present study, which included many patients whose average age was in the 70s and who were not in the later stages of life. In the EMPA-Elderly trial and other randomized controlled trials, the number of patients aged >75 years was small, which may not fully reflect the patient population in Japan and other super-aging societies. The results of the present study, which used real-world data, are more applicable to actual clinical conditions.

There is a reported trend in Japan of refraining from prescribing medications for guideline-directed medication therapy as people get older [9]. Practicing physicians feel that, regardless of the results of previous studies, there is concern about a situation in which the benefit outweighs the risk. The results of this study may be part of the evidence that practicing physicians’ concerns are appropriate and may help to inform whether SGLT2 inhibitors should be prescribed based on BI values in clinical information.

This study has some limitations. First, the generalizability of the results is limited due to the study's single-center observational design. Therefore, it is necessary to study this issue after recruiting more patients in multicenter studies. In addition, the number of patients in the frail SGLT2 inhibitor group was small, and the sample size was insufficient to detect HF-related events. Because it is not known whether the destination after treatment for HF exacerbations was in-home or institutional care, the impact of destination also could not be eliminated. Additionally, there was a number of patients for whom the BI was not measured; because this was a retrospective observational study, bias concerning factors affecting readmission could not be eliminated. Finally, we were not able to confirm medication adherence or drug continuation status, including interactions; therefore, we cannot rule out the possibility that these may have had an effect.

## Conclusions

This study suggests that the administration of SGLT2 inhibitors should be carefully considered for patients with HF with a BI <85 at the time of hospital discharge. Although SGLT2 inhibitors reduce the likelihood of HF-related rehospitalization in patients with low ADL, they appear to increase the overall risk of rehospitalization.
